# Editorial: Progress of programmed cell death in antitumor immunity

**DOI:** 10.3389/fimmu.2023.1230490

**Published:** 2023-06-12

**Authors:** Fangfang Yan, Chao Mao, Elaine Lai-Han Leung, Lianxiang Luo

**Affiliations:** ^1^ The First Clinical College, Guangdong Medical University, Zhanjiang, China; ^2^ Department of Experimental Radiation Oncology, The University of Texas MD Anderson Cancer Center, Houston, TX, United States; ^3^ Cancer Center, Faculty of Health Science, Ministry of Education (MOE) Frontiers Science Center for Precision Oncology, University of Macau, Macau, Macau SAR, China; ^4^ The Marine Biomedical Research Institute, Guangdong Medical University, Zhanjiang, China

**Keywords:** ferroptosis, cancer, immune checkpoint inhibitors, immunotherapy, tumor microenvironment, regulated cell death

Ferroptosis, a lipid peroxidative cell death that relies on iron, has been increasingly recognized. Ferroptosis can provide a new approach to anti-tumor therapy and reverse the resistance developed in cancer, which is a feasible therapeutic strategy (1, 2). Immunotherapy, designed to enhance a natural defense to eliminate malignant cells, is a great breakthrough in cancer therapy and has revolutionized the oncology field, reinvigorating the field of oncology immunology. Several types of immunotherapies, including adoptive cell transfer (ACT) and immune checkpoint inhibitors (ICIs), have achieved durable clinical responses, but their efficacy varies and only subpopulations of cancer patients can benefit (3). The tumor microenvironment refers to the noncancerous cells and their components present in the tumor, including the molecules that they produce and release. Immune infiltration in the tumor microenvironment (tumor microenvironment, TME) has been shown to play a key role in tumor development and will affect the clinical prognosis of cancer patients. The tumor microenvironment as a tumor therapeutic target has also attracted extensive research and clinical attention (4). Our Research Topic consists of two reviews and two original articles. Gu et al. found that in cancer treatment, glutathione, PUFAs, and other exogenous components involved in ferroptosis play an important role in regulating the sensitivity of target cells to ferroptosis; targeted therapy to suppress or induce ferroptosis may provide new options to improve the antitumor effect of immunotherapy strategies. Zhang et al. mentioned the developmental hierarchy of Tex for the effectiveness of adoptive T-cell metastasis and checkpoint blockade for cancer treatment during chronic infection and tumorigenesis, highlighting a prospective approach to improving the efficacy of ICB in cancer by exploiting the heterogeneity of T cells. The study of Dong et al. first constructed and validated prognostic features associated with ICd, including IFNB 1, IL 6, LY 96, and NT5E, to predict the survival and efficacy of immunotherapy in GC patients and found that this predictive model could well predict the outcome of immunotherapy treatment, providing new insights into effective individualized immunotherapy strategies. The study of Su et al. established and validated the RCD-Risk model based on Rcd-related genes, which predicted prognosis, TME, and immunotherapy outcomes in NSCLC patients well, and tissue microarray staining confirmed that the expression of the core gene LDLRAD3 in the RCD-Risk model was associated with poorer survival. They further explored the form of immunogenic cell death that can activate the adaptive immune response and further improves the effectiveness of immunotherapy for cancer and the uniqueness of ferroptosis. Interactions between cancer cells, immediate cells, and the tumor microenvironment are critical for simultaneous, offering new perspectives on differentiated approaches. This Research Topic effectively demonstrates the great potential of ferroptosis in cancer treatment and its ability to overcome the drug resistance effect generated in immunotherapy and improve the effectiveness of immunotherapy.

Ferroptosis plays an important role in modulating tumor immunity and immunotherapy response. Targeted ferroptosis alone or combination immunotherapy may offer new options to improve its antitumor efficacy. CD8 + T cells are essential for cytotoxic cells in antitumor immunotherapy. During tumorigenesis, these cells are often dysfunctional and present a “T cell” exhaustion, a unique state that is characterized by increased expression of inhibitory checkpoint receptors, and intervention against immune checkpoint blockade is considered a promising strategy to stimulate T-cell killing (5). Studies have shown that the metabolic pressure of mitochondria increases the ROS level of T cells under hypoxic conditions, leading to severe dysfunction and failure of T cells, thus reducing the internal ROS of T cells, alleviating the hypoxic environment of tumor cells, and effectively blocking T cell immune failure and synergistic anticancer effect with tumor immunotherapy (6). We received papers demonstrating that the development of Tex heterogeneity in tumors and chronic infections and the number of Tex intermediate subpopulations may be used as a predictor of inhibitory receptor blockade outcome, and understanding the developmental hierarchy of Tex has important implications for the effectiveness of adoptive t-cell metastasis and checkpoint blockade in treating cancer therapy. Oncogenesis is marked by a successful evasion of cell death regulation for unlimited replication and immortality. Activation of iron death may be a potential strategy to overcome the resistance mechanisms in conventional cancer therapies. Research has shown that target ferroptosis is expected to improve the efficacy of immunotherapy, representing a potential physiological function of ferroptosis in tumor suppression and immunosurveillance. These findings indicate the important role of ferroptosis in immunotherapy based on the interaction of ferroptosis with tumor immunotherapy, chemotherapy, and radiotherapy, thus indicating the significant potential of ferroptosis in cancer therapy (7, 8).

Immunogenic cell death (ICD) is a form of cell death that leads to the regulation and activation of the immune response and is characterized by the exposure and release of injury-related molecular patterns (DAMPs) in the tumor microenvironment (9). Studies have shown that the authors applied consensus clustering to obtain two ICD-related clusters of glioblastoma (GBM) and further constructed risk characteristics based on prognostic ICD genes. Based on the risk characteristics, a higher risk score was found to be associated with poorer patient outcomes. Iron death regulators/markers were highly enriched in the high-risk group, and ferroptosis was associated with cytokine signaling pathways and other immune-related pathways (10). These studies have been instrumental in developing and verifying prognostic characteristics related to ICD to anticipate survival and immunotherapy efficacy in cancer patients, thereby offering new insights into successful personalized immunotherapy approaches.

Modulating cell death (RCD) helps to reshape the tumor’s immune microenvironment (11). Studies have suggested that different forms of RCD may modify the tumor microenvironment (TME) by releasing pathogens or damage-related molecular patterns (PAMPs or DAAMPs), thereby affecting the benefits of anticancer therapy (12–14). Ferroptosis is immunogenic, and the unique metabolism and iron dependence of cancer cells suggest that they are more prone to ferroptosis through their interaction with cancer cells and play an important role in cancer treatment. The disorder of iron metabolism is considered one of the metabolic markers of malignant cancer cells. Iron is involved in the regulation of both innate and adaptive immune responses. In the process of the development of cancer, cancer cells’ iron-dependent metabolism makes ferroptosis a target for cancer; tumor cells’ sensitivity to ferroptosis, ferroptosis-related drugs (western medicine, Chinese medicine, and nanomedicine), and synthetic nanomaterials for ferroptosis was proved to have better lethality of cancer cells, making iron death occupy a place in immunotherapy and providing knowledge and ideas for the clinical value of cancer treatment strategy (15, 16). Ferroptosis-targeted therapy and immunotherapy may work together to create a powerful anti-tumor effect even for tumors that have been resistant to ICIs, providing a novel concept for further research and clinical use that could improve cancer treatment outcomes and patient prognosis. Yet, the exact mechanism and impact of ferroptosis on anti-tumor immunotherapy remain unclear and need to be explored in the future.

## Author contributions

LL and EL conceived and designed the editorial; FY wrote the editorial; CM, LL, and EL reviewed the paper and provided comments. All authors read and approved the final manuscript.
